# Anterior knee pain and sit-up tests predicts patients’ satisfaction and improvement in quality of life after anterior stabilized total knee replacement without patellar resurfacing

**DOI:** 10.1186/s40634-023-00641-9

**Published:** 2023-07-26

**Authors:** Maximiliano Barahona, Cristian Barrientos, Anselmo Alegría, Macarena A. Barahona, Tomas Navarro, Jaime Hinzpeter, Miguel Palet, Álvaro Zamorano, Jaime Catalán, Carlos Infante

**Affiliations:** Orthopaedic Department at Hospital Clinico Universidad de Chile, 999 Carlos Lorca Tobar Street, 3Rd Floor, Office 351. Independencia, Santiago, Chile

**Keywords:** Satisfaction, Total knee replacement, Total knee arthroplasty, Anterior knee pain, Patellar resurfacing, Patellar preservation, Anterior stabilized

## Abstract

**Purpose:**

The purpose of this study was to assess patient satisfaction and identify risk factors for dissatisfaction after anterior stabilised conventional total knee arthroplasty (TKA) without patellar resurfacing, using the Goodman score.

**Methods:**

We conducted a cross-sectional study using data from our institutional database from 1 January 2018 to 1 March 2021. Patients who underwent TKA with the Vanguard® Cruciate Retaining Anterior Stabilized Knee System (Zimmer Biomet, Warsaw, Indiana, USA) without patellar replacement were included. Patients with other bearing surfaces (posterior stabilised or medial congruent) or diagnosed with infection or instability were excluded. Patients' reported outcomes, body mass index (BMI), passive range of motion, the timed up-and-go test, sit-up test, and algometry were assessed. Patients were also asked if they had anterior knee pain. Satisfaction was assessed using the Goodman scale, and logistic multivariate regression was used to identify variables associated with dissatisfaction and perceived improvement in quality of life.

**Results:**

A total of 131 TKA patients were included in the study. The median satisfaction score was 100 (interquartile range [IQR], 87.5 to 100), with the 75-point threshold at the 90th percentile according to Section A of Goodman. Section B of Goodman showed that 113 TKA patients (86.26%) reported "great improvement" or "more than I ever dreamed." Multivariate logistic regression revealed that anterior knee pain (OR 5.16, 95% CI 1.24 to 21.39), the sit-up test (OR 0.63, 95% CI 0.49 to 0.81), and BMI (OR 0.84, 95% CI 0.70 to 0.99) were significantly associated with patient dissatisfaction and a worse perceived improvement in quality of life. The receiver operating characteristics curve for the models had areas under the curve of 0.83 (95% CI 0.69 to 0.97) and 0.82 (95% CI 0.70 to 0.94), respectively.

**Conclusion:**

Anterior stabilised TKA without patellar resurfacing can achieve 90% satisfaction and 86% improvement in quality of life. To improve these results, it is essential to prevent and treat anterior knee pain and enhance quadriceps strength.

**Level of evidence:**

Level III (retrospective cohort study).

## Introduction

Total knee arthroplasty (TKA) is a common procedure aiming to restore function and alleviate pain in patients with end-stage knee osteoarthritis [48]. The impact of this procedure on quality of life has led to the adoption of the number of TKAs per 100,000 inhabitants as a quality indicator of health standards by the Organisation for Economic Co-operation and Development (OECD) [46].

Patient satisfaction and improvement in quality of life are the primary outcomes to be achieved after TKA. Recently, Goodman et al. developed a reliable scale to objectively measure both outcomes, which has been validated and adapted for Spanish speakers [7, 18]. Historically, dissatisfaction rates were set at 20% [31, 55]. However, advancements in pain management, implant technologies, and surgical understanding have raised satisfaction levels to nearly 90%, according to recent reports [12].

Currently, numerous technical controversies exist, such as the choice of polyethylene insert and the necessity of performing patella resurfacing [57]. Many studies have shown no significant differences in anterior knee pain incidence, functional outcomes, and range of motion (ROM) after patellar resurfacing [19, 21]. Additionally, the likelihood of reducing knee pain after TKA by performing secondary patellar replacement is similar to chance [49]. The advantages of not replacing the patella include preserving bone stock, avoiding patellar complications related to cuts—such as fracture or malalignment of the insert—and saving time [49]. Recent literature has not demonstrated a significant difference between cruciate retaining (CR) or posterior stabilised (PS) techniques, leaving the decision to the surgeon's preferences [51].

Our institution is a university hospital that led the TKA volume in our country from 2004 to 2019 [4]. The most common TKA procedure in our university hospital involves not replacing the patella and using a CR anterior stabilised insert. At present, the quality and quantity of satisfaction reports after TKA are limited, with study heterogeneity [26] complicating data pooling, making it difficult to extrapolate these results to all TKAs. The purpose of this study was to determine satisfaction following an anterior stabilized conventional total knee replacement without patellar resurfacing, using the Goodman score, and to identify risk factors for dissatisfaction after TKA. We hypothesize that this cohort will achieve a higher level of satisfaction than the historical average of 80%, and we anticipate identifying at least one factor that can predict satisfaction.

## Materials and methods

A cross-sectional study was designed and approved by our local ethics committee and was conducted in accordance with the declaration of Helsinki. The institutional database was reviewed from 1 January 2018 to 1 March 2021. Patients were invited to participate in the study if they had undergone TKA using the Vanguard® Cruciate Retaining Anterior Stabilised Knee System (Zimmer Biomet, Warsaw, Indiana, USA) without patellar replacement. Patients were excluded if another bearing surface was used (PS or medial congruent) or if they had a diagnosis of infection or instability. TKA was performed using a medial parapatellar approach and eversion of the patella. Neither a tourniquet nor wound drainage was used. Extra or intramedullary guidance for tibial alignment was used according to the surgeon's preference, while intramedullary guidance for femoral alignment was used in all cases. The epicondylar axis and the Whiteside line were used for rotational alignment on the femoral component; conversely, the posterior cruciate ligament to tibial tubercle axis and the anterior cortex were used as reference points for rotation on the tibia. The surgeons in this study never replaced the patella and always performed patellar denervation.

**Fig. 1:**
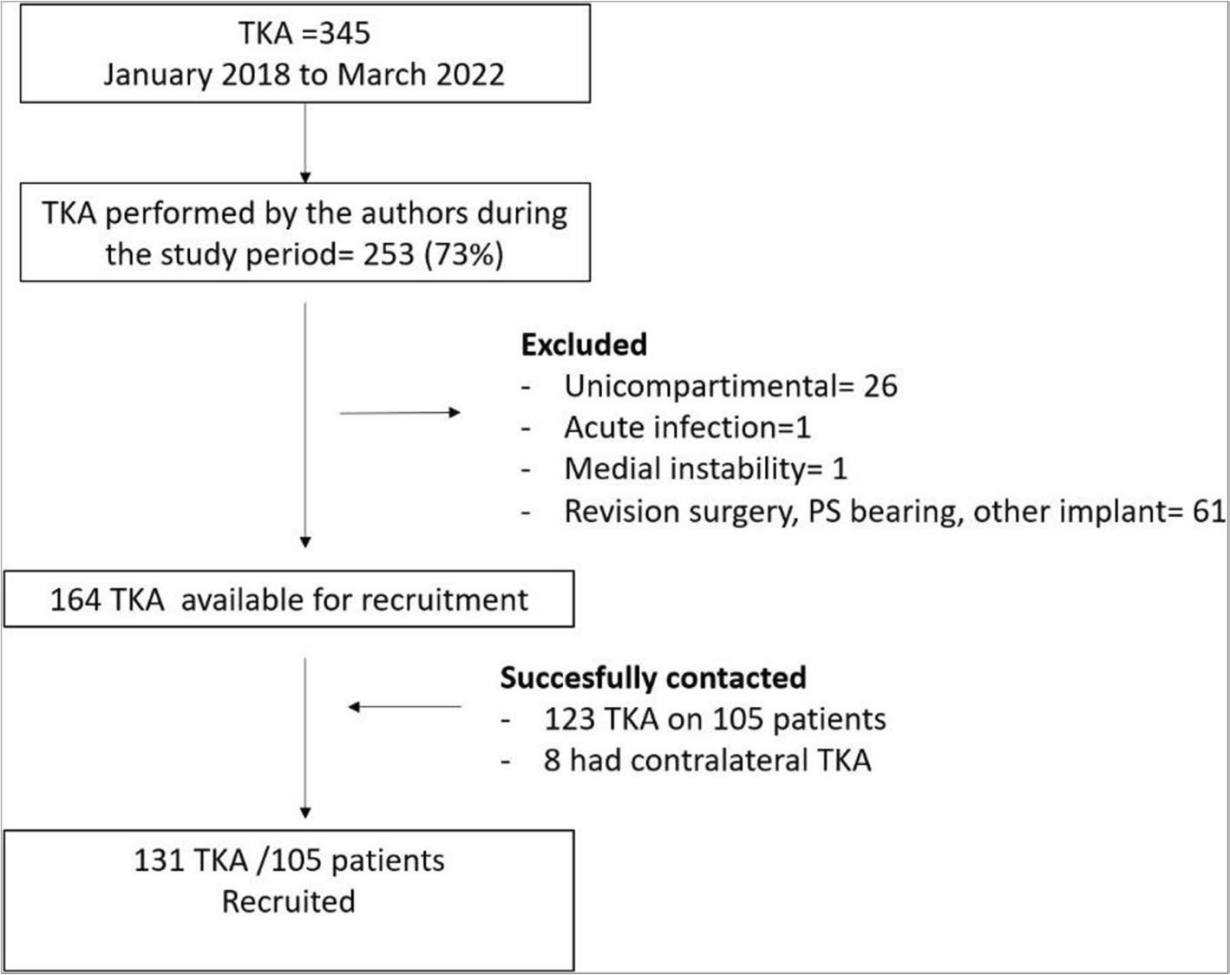
Flow chart. Abbreviations: TKA, total knee arthroplasty; PS, posterior stabilised

A total of 345 TKAs were identified. The four surgeons who participated in the study carried out 253 of them (73%). Twenty-six of the 253 (10%) were unicompartmental arthroplasties, and 61 (24%) TKAs were not included because they were revision surgeries, or a PS bearing was used. One hundred sixty-six TKAs were identified for recruitment, of which one patient was excluded for acute infection (0.6%) and one (0.6%) for medial instability. Finally, 123 of 164 TKAs (75%), corresponding to 105 patients, were successfully contacted and agreed to participate in the study. All of them received counselling and then signed written informed consent to participate. If a patient had a contralateral TKA performed before the time frame of this study at our institution using the same model, both TKAs were recruited. This occurred in eight patients (8/105; 7.6%), totalling 131 recruited TKAs in 105 patients (Fig. 1).

All evaluations were performed by the same physiotherapist (MaB) from March 2022 to September 2022. The assessment included patients' reported outcomes, body mass index (BMI), passive ROM, the timed up-and-go (TUG) test, sit-up test, and algometry. Additionally, all patients were asked if they experienced AKP during their daily activities, regardless of intensity and frequency (daily or weekly). The response was recorded as either yes or no.

The principal outcome of the study was satisfaction and patients' perceived improvement in their quality of life after TKA, using the Goodman scale, which has been validated for TKA and adapted for Spanish speakers [7, 18, 56]. This scale has two sections. The first section ("A") aims to evaluate satisfaction using four questions ranging from 0 "very dissatisfied" to 4 "very satisfied." These four questions can be summarised by calculating the average score and multiplying by 25, with the minimum score being 0 "very dissatisfied" and the maximum score 100 being "very satisfied." A score of at least 75 was considered satisfactory as it represents an average of 3 on the four questions. The second section ("B") aims to evaluate the degree of improvement perceived by the patient compared to their pre-surgery status. This item is a Likert scale from 1 "worst" to 6 "better than I ever dreamed." Furthermore, patients were asked to complete the Western Ontario and McMaster Universities Arthritis Index (WOMAC), Kujala, and Knee Injury and Osteoarthritis Outcome Score (KOOS) questionnaires to assess their quality of life.

The passive ROM was assessed with the participant in the supine position, with hips in the neutral position. A 360° universal plastic goniometer with a 30-cm movable arm and a scale of 1° increments (Baseline®, Chattanooga Group, Inc, Hixson, Tennessee, USA) was used for all measurements. The stationary arm was placed along the femur to the greater trochanter. The movement arm was placed along the fibula to the lateral malleolus. The fulcrum was visually positioned at the trans epicondylar axis of the knee joint. For knee flexion, the heel of the foot was required to be in contact with the examination couch during the assessment. For passive knee extension, a cylindrical roll was placed underneath the heel of the foot to allow the knee to extend as much as possible. Both knees were assessed [47].

The TUG test corresponds to the time it takes for a patient to get up from a chair, walk 1.5 m in a straight line, turn around, and return (covering a total distance of 3 m). The time was recorded in seconds [22]. For the sit-up test, the patient was asked to complete as many sit-and-stand cycles as quickly as possible in 30 s with their arms crossed over their chest. Then, the muscle power was estimated using a previously published formula. [2, 45]: $\frac{Weight \left(kg\right)* 0.9 * 9.81 *\left(\left(height\left(mts\right)*0.5\right)-"chair\,height\left(mts\right)"\right)}{\left(\frac{30}{number}\,of\,repetitions\right)*0.5}.$ The algometry, the patient was asked to kneel over a weighting machine three times, and the maximum kilograms that the patient tolerated was recorded and compared with the contralateral knee.

Continuous variables were summarised as median (50th percentile), range, and IQR (25th percentile to 75th percentile). Categorical variables were summarised using frequency and percentages. A comparison was performed for all variables between patients with one-sided TKA and those with bilateral TKA.

The Goodman scale was the primary outcome [20]. A quantile regression was estimated to determine the variables that could predict the score of Section A. The pseudoR2 was used to determine the ability to predict. For those variables with a significant coefficient (p < 0.05) and a pseudoR2 above 0.15, a bootstrapped multivariate quantile regression with 200 repetitions using stepwise was estimated. Additionally, the score was dichotomised using the score 75 as the threshold, and a multivariate logistic regression was estimated. After logistic regression, the goodness of fit test was performed to determine if the model was adequately estimated.

Section "B" was dichotomised into two categories for analysis purposes: answers from 1 to 4 were classified as "Same or Worse", and answers from 5 to 6 as "Much better". Then, a logistic regression was estimated to determine risk factors for "Same or Worse". For those variables with a significant odds ratio (OR), the area under the receiver operating characteristic (ROC) curve was estimated to evaluate the ability to discriminate. A bootstrapped multivariate logistic regression (MLR) with 200 repetitions using stepwise was performed. After the MLR, the goodness of fit test, with a maximum of 10 group covariances, was conducted to determine if the model was adequately estimated.

## Results

A total of 131 TKAs were recruited from 105 patients, of whom 46 were men (43.81%). The median age at surgery was 66 years (range, 47 to 88; IQR, 62 to 71), 78 TKAs were on the right side (59.54%), and 13 patients underwent bilateral TKA. The median follow-up was 2.74 years (range, 1.01 to 5.1; IQR, 1.71 to 3.85).

No clinically relevant difference in functional evaluation was found between patients with one-sided TKA and bilateral TKA (Table 1); thus, no inferential statistics were performed. The median ROM was 115, with a median difference of -5 degrees to the contralateral side, which increased by five degrees more in the case of unilateral TKA (Table 1). Two patients required a second intervention; one underwent mobilisation under anaesthesia and arthroscopic debridement for arthrofibrosis, with a final ROM of 100 degrees and a side-to-side difference of 28 (unilateral TKA); their satisfaction was 100 (Goodman A) and improvement 6 ("most I ever dreamed"). The other required selective embolisation for recurrent hemarthrosis and reported a score of 100 in Goodman A and 5 in Section B.

**Table 1:** Clinical evaluations for the total cohort, one-sided TKA, and bilateral TKA

	Total patients	One-side TKA	Bilateral TKA
Flexion	112 (82 to 135) (106 to 120)	112 (82 o 135) (106 to 120)	111 (90 to 127) (105 to 119)
-Diff	-5 (-42.3 to 21) (-12 to 2)	-10.4 (-42 to 21) (-14 to -4)	N/A
EXT	2 (-12 to 16) (0 to 6)	2 (-6 to 16) (0 to 6)	3 (-12 to 15) (0 to 6)
-Diff	0 (-12 to 16) (-2 to 4)	2 (-8 to 16) (0 to 6)	N/A
ROM	115 (80 to 147)	116 (80 to 147) (108 to 124)	115 (91 to 132) (107 to 122)
-Diff	-5 (-44 to 25)	-9 (-44 to 25) (-15 to 3)	N/A
Algometry	11 (0 to 36) (7 to 14)	10 (0 to 25) (6 to 15)	11 (3 to 37) (8 to 14)
-Diff	-2 (-16 to 10) (-6 to 1)	-4 (-17 to 10) (-8 to 1)	N/A
Sit-up test	14 (3 to 27) (11 to 15)	14 (3 to 27) (12 to 15)	14 (3 to 21) (11 to 15)
	218 (43 to 405) (172 to 268)	222 (79 to 405) (172 to 268)	217 (43 to 390) (177 to 268)
TUG	10 (6 to 33) (8 to 11)	9 (6 to 23) (8 to 11)	9 (6 to 33) (8 to 11)
BMI at evaluation	31 (21 to 44) (28 to 32)	31 (21 to 44) (27 to 33)	31 (25 to 43) (29 to 33)

*Abbreviations*: *TKA* Total knee arthroplasty, *TUG* Timed up-and-go test, *BMI* Body mass index, *ROM* Range of motion, *EXT* Extension

Algometry showed a side-to-side median difference of 2 kg, which increased to a median of 4 kg in unilateral cases. The median sit-up and TUG tests were 14 sit-ups and 10 s, respectively. The BMI at follow-up ranged from 21 to 44 kg/m^2^, with 55 patients (42%) between 30 to 35 kg/m^2^ and 20 patients (15%) above 35 kg/m^2^ (Table 1). Anterior knee pain was reported by 27% (95% Confidence Interval [95%CI], 19% to 35%) of the included TKAs; no clinical difference between one-sided TKA and bilateral TKA was found, reaching 26% (95% CI, 15% to 41%) and 27% (95% CI, 18% to 38%), respectively. All patients' reported outcomes are summarised in Table 2.

**Table 2:** Patient reported outcomes

	Median (Range)	IQ range
Goodman A	100 (0 to 100)	87.5 to 100
Goodman B	5 (1 to 6)	5 to 6
WOMAC-pain	2 (0 to 15)	1 to 5
- Normalized	0.1 (0 to 0.75)	0.05 to 0.25
WOMAC-stiffness	1 (0 to 8)	0 to 2
- Normalized	0.13 (0 to 1)	0 to 0.25
WOMAC-Function	9 (0 to 55)	4 to 19
- Normalized	0.13 (0 to 0.81)	0.06 to 0.30
Kujala	75 (20 to 100)	63 to 86
KOOS QL	62.5 (5 to 100)	43.75 to 81.25

*Abbreviations*: *WOMAC* Western Ontario and McMaster Universities Osteoarthritis Index, *KOOS QL* Knee injury and osteoarthritis outcome score quality of life, *IQ* Interquartile

The median satisfaction was 100 (IQR, 87.5 to 100), according to Section A of the Goodman scale (Table 2). The 75-point threshold was at the 90th percentile, with only seven cases reporting a score of 50 or lower. The lowest satisfaction percentage (20 TKAs, 15.3%) was achieved in question 3 of Section A, which asked about satisfaction with performing physical activities (Table 3). In Section B of the Goodman scale, 113 TKAs (86.26%) reported "great improvement" or "more than I ever dreamed"; conversely, 18 (patients 13.74%) rated their improvement as moderate or less (Table 4).

**Table 3:** Distribution of answers of Section A of the Goodman scale by each question

Satisfaction with	Very Unsatisfied	Somewhat Unsatisfied	Neither	Somewhat satisfied	Very satisfied
Pain relief	3 (2.3%)	1 (0.8%)	4 (3.1%)	17 (12.98%)	106 (80.9%)
Ability to do house/yard work	2 (1.5%)	0	8 (6.1%)	26 (19.9%)	95 (72.5%)
Ability to do recreational activities	4 (3.1%)	4 (3.1%)	12 (9.2%)	37 (28.2%)	74 (56.5%)
Overall Satisfaction	2 (1.5%)	0	8 (6.1%)	15 (11.5%)	106 (80.9%)

**Table 4:** Frequency of answers in the B section of the Goodman Scale (patient-perceived improvement)

Goodman B	Frequency		Frequency
Worse	1 (0.76%)	Same or worse	18 (13.74%)
Same as before	5 (03.82%)		
Low improvement	6 (04.58%)		
Moderate improvement	6 (04.58%)		
Great improvement	74 (56.49%)	Much Better	113 TKA (86.26%)
More than I ever dreamed	39 (29.77%)		

*Abbreviation*: *TKA* Total knee arthroplasty

**Fig. 2:**
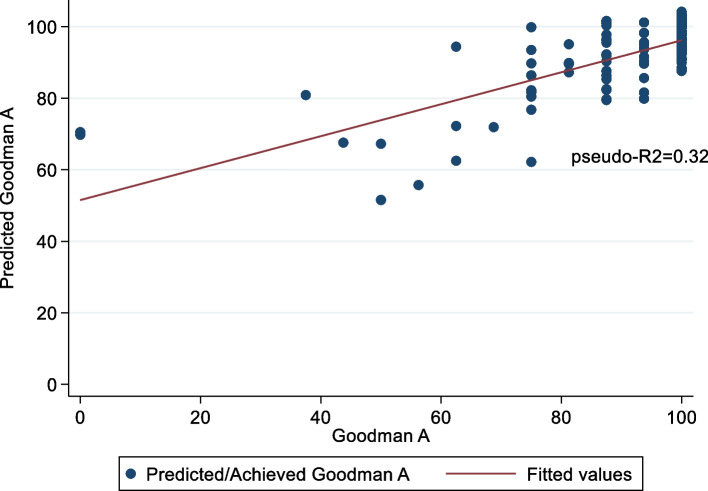
A scatter plot between the predicted score and the actual score of Section A of the Goodman scale. The red line corresponds to the fitted linear prediction

**Fig. 3:**
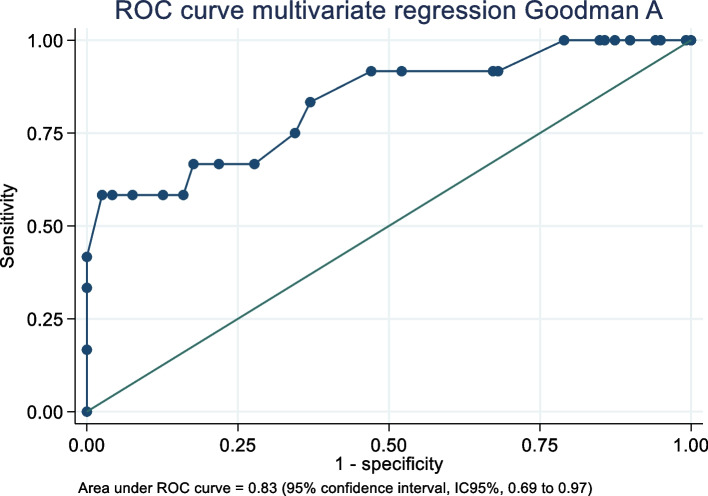
The ROC curve was estimated to determine the model's discrimination for Section A of the Goodman scale. The independent variables were anterior knee pain and the number of sit-ups performed in 30 s at the final follow-up. Abbreviations: ROC, receiver operating characteristics

The quantile regression estimated that no functional evaluation—ROM, sit-up, or TUG—nor BMI had a significant association with patient satisfaction (Goodman A). Anterior knee pain and gender showed a significant coefficient but with a low pseudo R2. Conversely, patient-reported outcomes demonstrated a significant coefficient with pseudo-R2 ranging from 0.18 to 0.26. Multivariate bootstrapped quantile regression indicated that the best-predicted model was the pain and stiffness WOMAC and the Kujala score (Table 5). The predicted Goodman A (PGoodmanA) was estimated as follows: *PGoodmanA* = 88.14103 + (-1.602564 * WOMAC A) + (-2.350427 * *WOMAC B*) + (0.1602564 * *Kujala*), reaching a pseudo-R2 of 0.32. The scatter plot (Fig. 2) shows that the model fails to predict the lowest scores of Goodman A but provides a good estimation for scores above 40 points. Conversely, when the score was dichotomised using 75 points as the threshold, an MLR demonstrated that anterior knee pain (OR 5.16, 95% CI 1.24 to 21.39) and sit-up test (OR = 0.63, 95% CI 0.49 to 0.81) were significant. The area under the ROC curve was 0.83 (95% CI, 0.69 to 0.97), and the goodness-of-fit test had a probability of 0.71 (Fig. 3).

**Table 5:** Coefficient, *p*-value and pseudo-R2 after univariate quantile regression to predict the Goodman A score

Variable	Coefficient	*P*-Value	Pseudo-R2
Age	0 (-0.32 to 0.32)	0.999	0.001
Male	-6.25 (-11.94 to -0.56)	0.030*	0.001
WOMAC-pain	-2.84 (–3.33 to -2.36)	< 0.000*	0.260
WOMAC-Stiffness	-6.25 (-7.43 to -5.07)	< 0.000*	0.240
WOMAC-Function	-0.80 (-0.95 to 105.03)	< 0.000*	0.240
Kujala	0.42 (0.26 to 0.59)	< 0.000*	0.200
KOOS QL	0.22 (0.12 to 0.32)	< 0.000*	0.180
Anterior knee pain	-12.5 (-20.6 to -4.41)	0.003*	0.060
Follow-up	-0.70 (-2.51 to 1.11)	0.444	0.010
ROM	0 (-0.21 to 0.21)	0.999	0.001
Extension	0 (-0.61 to 0.61)	0.999	0.001
Extension difference	0 (-0.61 to 0.61)	0.999	0.001
Flexion	0 (-0.25 to 0.25)	0.999	0.001
Flexion difference	0 (-0.24 to 0.24)	0.999	0.001
BMI at FU	0 (-0.59 to 0.59)	0.999	0.001
Up and Go test	-0.64 (-1.32 to 0.05)	0.070	0.020
Situp test	0 (-0.81 to 0.81)	0.999	0.001
Muscular force	0 (-0.03 to 0.03)	0.999	0.001
Algometry	0.32 (-0.12 to 0.77)	0.150	0.002

*Abbreviations*: *WOMAC* Western Ontario and McMaster Universities Osteoarthritis Index, *KOOS QL* Knee injury and osteoarthritis outcome score quality of life, *ROM* Range of motion, *BMI* Body mass index, *FU* Follow-up

^*^significant coefficient (*p*-value < 0.05)

**Fig. 4:**
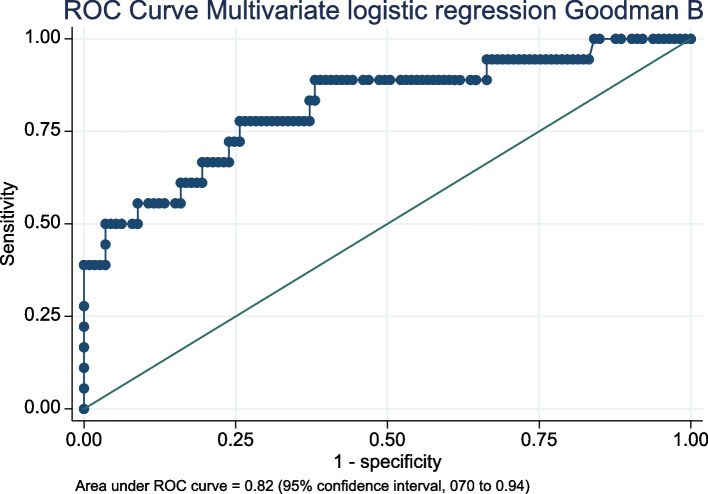
The ROC curve of the bootstrapped multivariate logistic regression was estimated to predict Section B of the Goodman scale. The independent variables were the presence of anterior knee pain, BMI, and the number of sit-ups performed in 30 s at the final follow-up. Abbreviations: ROC, receiver operating characteristics; BMI, body mass index

Logistic regression showed a significant association between the perceived improvement by patients (Goodman B) and patient-reported outcomes, anterior knee pain, years at follow-up, BMI at follow-up, sit-up, and TUG tests (Table 6). MLR indicated that anterior knee pain, sit-up test, and BMI were associated with patient-perceived improvement (Goodman B). Patients with anterior knee pain showed an odds ratio (OR) of 4.29 (95%CI, 1.20 to 15.30), BMI exhibited an OR of 0.84 (95%CI, 0.70 to 0.99), and sit-up was 0.74 (95%CI, 0.57 to 0.97), meaning that patients without anterior knee pain, higher BMI, and better performance in the sit-up test were more likely to perceive a better impact after TKA. The area under the ROC curve was 0.82 (95%CI, 0.70 to 0.94), and the goodness-of-fit test had a probability of 0.16 (Fig. 4). Multivariate models using patient-reported outcomes did not significantly improve the area under the ROC curve.

**Table 6:** Comparison of patient-reported outcomes and functional evaluation among those patients who scored 5 or 6 (“Much better”) on Section B of the Goodman scale and those scoring 4 or less (“Same or worse”)

Variable	Much better	Same or worse	Odd Ratio	AUC
Age at surgery	65 (48 to 86) (62 to 70)	70 (47 to 88) (62 to 77)	1.04 (0.98 to 1.11)	N/A
Male	49 (43%)	9 (50%)	1.31 (0.48 to 3.54)	N/A
WOMAC-pain	2 (0 to 8) (1 to 3)	10 (2 to 15) (6 to 14)	1.98 (1.47 to 2.68)*	0.94 (0.87 to 0.99)
WOMAC-Stiffness	1 (0 to 3) (0 to 2)	3 (0 to 8) (2 to 5)	3.00 (0.01 to 0.07)*	0.83 (0.71 to 0.95)
WOMAC-Function	7 (0 to 36) (4 to 14)	30 (11 to 55) (26 to 44)	1.17 (1.10 to 1.25)*	0.93 (0.87 to 0.98)
Kujala	77 (32 to 100) (66 to 87)	43 (29 to 78) (34 to 60)	0.89 (0.85 to 0.93)*	0.91 (0.84 to 0.98)
KOOS QL	69 (5 to 100) (50 to 81)	34 (12 to 87.5) (18.75 to 50)	0.95 (0.92 to 0.97)*	0.81 (0.69 to 0.93)
Anterior knee pain	25 (22%)	10 (56%)	4.4 (1.57 to 12.33)*	0.67 (0.54 to 0.79)
Follow-up	2.7 (1 to 5) (2 to 4)	3 (1 to 4) (2 to 4)	1.03 (0.77 to 1.40)*	0.58 (0.44 to 0.72)
ROM	115 (91 to 147) (107 to 123)	116 (80 to 132) (109 to 123)	0.98 (0.94 to 1.02)	N/A
Extension	2 (-6 to 16) (0 to 6)	2 (-12 to 12) (0 to 6)	0.92 (0.82 to 1.03)	N/A
Extension difference	2 (-12 to 16) (-2 to 4)	0 (-8 to 12) (4 to -4)	1.03 (0.93 to 1.15)	N/A
Flexion	112 (89 to 135) (106 to 120)	113 (82 to 127) (109 to 123)	0.99 (0.94 to 1.04)	N/A
Flexion difference	-5 (-33 to 21) (-12 to 2)	-8 (-42 to 17) (-12 to 0)	1.02 (0.97 to 1.06)	N/A
BMI at FU	31 (21 to 44) (29 to 33)	28 (23 to 37) (25 to 31)	0.83 (0.72 to 0.97)*	0.70 (0.56 to 0.83)
Up and Go test	9 (6 to 26) (8 to 10)	12 (6 to 33) (8 to 14)	1.16 (1.05 to 1.28)*	0.69 (0.52 to 0.86)
Sit-up test	14 (7 to 27) (12 to 15)	11 (3 to 19) (9 to 13)	0.74 (0.62 to 0.88)*	0.73 (0.58 to 0.88)
Muscular force	222 (94 to 405) (185 to 290)	177 (43 to 313) (118 to 246)	0.98 (0.98 to 0.99)*	0.69 (0.55 to 0.84)
Algometry	11 (2 to 37) (7 to 15)	8 (0 to 22) (5.4 to 12)	0.91 (0.82 to 1.01)	N/A

The OR was estimated using univariate logistic regression to predict whether to be in the same or worse group. The AUC was estimated when the OR was significant

*Abbreviations*: *WOMAC* Western Ontario and McMaster Universities Osteoarthritis Index, *KOOS QL* Knee injury and osteoarthritis outcome score quality of life, *ROM* Range of motion, *BMI* Body mass index, *FU* Follow-up, *OR* Odds ratio, *AUC* Area under the curve

^*^significant coefficient (*p*-value < 0.05)

## Discussion

The main finding of this study was that 86% of patients reported significant improvement, and 90% reported at least 75 points out of 100 in satisfaction after TKA with anterior stabilisation insert and no patellar resurfacing. Dissatisfaction after TKA has been historically documented to be around 20% [20]. A recent study showed dissatisfaction to be 22.2% and identified several risk factors: residual pain, female gender, primary diagnosis, ROM after surgery, and wound healing [44]. Nevertheless, other studies have recently reported that satisfaction rates are increasing after TKA, with rates nearing 90% achieved, as in this current study [12].

The main reasons for improved satisfaction after TKA are better implant design, increased understanding of TKA biomechanics and patellar tracking, and advancements in perioperative pain management [17, 38, 42, 54]. The recent development of better instruments to assess satisfaction has enabled researchers to compare more reliable results and establish what satisfaction means for patients [27]. The Goodman scale is reliable and aims to determine satisfaction and patients' self-perceived improvement after TKA [7, 18, 56].

Knee anterior pain and performance in the sit-up test were significantly associated with satisfaction and the improvement perceived by patients. This is consistent with other studies, as patients' expectations before surgery are a well-documented risk factor for dissatisfaction [39]. These expectations are mainly related to pain relief and function [35, 36].

We report a relatively high incidence of anterior knee pain after AS TKA, but within the range found in the literature [37, 43]. A simple explanation for anterior knee pain could be patellar preservation. Nevertheless, the incidence of anterior knee pain has been reported to be no different in cases of patellar resurfacing, due to component alignment in the axial patellar view [29, 40]. In the case of patellar retaining TKA, a more trochlear-friendly design has been shown to decrease the incidence of anterior knee pain [14, 32]. Moreover, a better understanding of the patellofemoral relationship in TKA is paramount: femoral offset, sagittal alignment, and rotation are essential for improving results in TKA without patellar resurfacing [1, 28, 29]. Roessler et al. reported that tibial component rotation was an important factor in predicting which TKA would require secondary patellar resurfacing [50]. Future studies must relate anterior knee pain to radiological parameters.

Additionally, the type of bearing surface seems to be related to anterior knee pain, with secondary patellar replacement being performed more frequently on PS cases, according to the German registry [5]. Nevertheless, meta-analyses have shown no difference in the prevalence of anterior knee pain between PS or CR TKA designs [25, 34].

A recent study comparing TKA with patellar resurfacing and TKA with patellar denervation found that denervation decreased the intensity of anterior knee pain the most, with similar satisfaction among groups at 24-month follow-ups [24]. Denervation is routinely performed in our TKA and could explain why some patients are satisfied despite residual anterior knee pain. However, other studies suggest a diminishing effect on anterior pain with denervation as the follow-up increases [59]. Another explanation for patients being satisfied despite anterior knee pain is that having anterior knee pain before surgery is a risk factor for experiencing it after surgery [17, 53]. Thus, surgery may not eliminate the pain, but if the intensity of pain is significantly decreased, patients might report satisfaction.

Infection and malalignment should be ruled out in cases of anterior knee pain after surgery. Subsequently, an interdisciplinary approach must be taken for pain management [13, 41]. Radiofrequency ablation of the genicular nerves has been proposed for treating anterior knee pain in patients with osteoarthritis, mainly in patients with a high perioperative risk [6]. This interventional technique could also maximise results after TKA, especially in those with a documented neuroma [15], but also in patients unsatisfied by anterior knee pain without a clear cause. In the latter, a previous blockage of the genicular nerves could be used as a diagnostic test to proceed further with the ablation [10].

Performance in TUG, muscular force, and the sit-up test were significantly associated with the patient's perception of improvement. The latter was also significant in the multivariate analysis for both sections of the Goodman scale. All these tests indirectly assess quadriceps strength, which is impaired after TKA compared to age-matched controls. Furthermore, low quadriceps strength before surgery has been associated with a longer period of recovery after TKA [23]. No specific muscle strength programme has shown superiority over others [3, 16, 52], but it seems that an exercise programme should be continued long-term after TKA [23]. This cohort had undergone heterogeneous exercise programmes after TKA, and those who performed better on the performance test were associated with greater satisfaction and perceived outcome.

Quadriceps strength and anterior knee pain are well-known related problems as well [30]. Anterior knee pain might lead patients to avoid strengthening programmes, leading to quadriceps atrophy which increases anterior pain [33]. This relationship has been found to be significant in patients after TKA [8], which might explain the association of anterior knee pain and the sit-up test found in the multivariate analysis of this study.

Another finding in our report was that patients with greater BMI had a significant trend to self-perceive a more remarkable improvement after TKA, but similar satisfaction. Contradictory statements are found in the literature on this topic [11]. Our results could be explained because the status before surgery in patients with greater BMI was worse than those with lesser BMI [58]. Therefore, patients with increased BMI should be given appropriate advice on their increased risk of infection and other complications [9]; however, both the surgeon and the patients should be aware that these patients are more likely to improve after surgery. Also, patients tend to increase their weight after surgery, according to the literature [11], so this finding could be interpreted as indicating that although patients gain weight after surgery, they can expect improvement compared to their pre-surgery status.

Finally, WOMAC, Kujala, and KOOS quality of life were strongly related to the Goodman scale, which makes our results more reliable and not biased by one patient-reported outcome. Also, it could help other surgeons to estimate their patients' satisfaction.

The main limitation of our study is the low sample size compared to other studies. Nevertheless, the findings consistently show that the same variables—anterior knee pain and sit-up test—were related to satisfaction and patients' improvement, and the bootstrapped regression accounts for this limitation. Also, these findings cannot be instantaneously extrapolated to other types of insert or patellar resurfacing. Additionally, this study did not use radiological assessment, which may bias the results. A further step in our research is determining which radiological parameters predict anterior knee pain in TKA without patellar resurfacing.

## Conclusions

Anterior stabilised TKA without patellar resurfacing can achieve 90% satisfaction and 86% improvement in quality of life. To improve these results, it is essential to prevent and treat anterior knee pain and enhance quadriceps strength.

## Data Availability

The datasets used or analyzed during the current study are available from the corresponding author on reasonable request and approval from the ethical board commitee of our institution.

## Abbreviations

95%CI: 95% Confidence Interval
BMI: Body Mass Index
CR: Cruciate Retaining
IQR: Interquartile Range
KOOS: Knee injury and Osteoarthritis Outcome Score
MLR: Multivariate Logistic Regression
OR: Odds Ratio
PS: Posterior Stabilised
ROC: Receiver Operating Characteristic
ROM: Range of Motion
TKA: Total Knee Arthroplasty
TUG: Timed Up and Go
WOMAC: Western Ontario and McMaster Universities Osteoarthritis Index
